# The effect of Er, Cr:YSGG laser irradiation on the apical leakage of retrograde cavity

**Published:** 2009-10-10

**Authors:** Mohammad Asnaashari, Reza Fekrazad, Fatemeh Dehghan Menshadi, Massoud Seifi

**Affiliations:** 1 *Department of Endodontics, Iranian Center for Endodontic Research, Dental School, Shahid Beheshti University of Medical Sciences, Tehran, Iran*; 2 *Department of Periodontist, Dental School, Shahid Beheshti University of Medical Science, Tehran, Iran*; 3 *Department of Endodontics, Dental School, Zahedan University of Medical Sciences, Zahedan, Iran*; 4 *Department of Orthodontics, Dental School, Shahid Beheshti University of Medical Sciences, Tehran, Iran*

**Keywords:** Apical seal, Debris, Er, Cr:YSGG laser, Smear layer

## Abstract

**INTRODUCTION:** Controversial results have been reported when organic acids, ultrasonic instruments and laser techniques were used to remove smear layer in endodontic treatments. The aim of this study was to evaluate the effect of removing debris and smear layer by Er,Cr:YSGG laser irradiation on the apical leakage of retrograde cavities.

**MATERIALS AND METHODS:** In this *ex vivo* study, 24 extracted mandibular single-rooted teeth were selected and instrumented up to K-file size #35. Approximately 3 mm of root apices were dissected perpendicular to the root’s long axis. Retrograde cavities with 3 mm depth were prepared and the teeth were randomly assigned to two groups. In one group, the retrograde cavities were filled with amalgam and in the other group, the dentinal surface of the retrograde cavities were lased with Er,Cr:YSGG laser (2W, 15 seconds, G4 tip). The cavities were filled with amalgam; all tooth surfaces except for dissected outsides were covered with blue wax. Then the teeth were immersed in 2% methylene blue dye for 48 hours. The amount of dye penetration into sagittal sections was measured by stereomicroscope at ×20 magnification by two independent observers who were blinded to the experiment. Data were statistically analyzed using student t-test.

**RESULTS:** This study demonstrated that dye penetration was 0.8 mm (±0.53) in the lased and 0.97 mm (±0.54) in the non-lased group. It showed that, Er,Cr:YSGG laser can remove the debris and smear layer and consequently reduces the amount of dye penetration, although, the difference between the two groups was not statistically significant.

**CONCLUSION:** This study showed that dye penetration was less in lased group because of the better seal of the dissected surface due to the better removal of the debris and smear layer by laser; further investigations are recommended in this field. [Iranian Endodontic Journal 2009;4(4):144-8]

## INTRODUCTION

Failures in apicectomy are mostly due to the permeability of dentinal surface and inadequate sealing of retrofilling material. This will lead to the percolation of microorganisms and their products from the root canal system into the dentinal tubules and periodontal region (1,2). Therefore, the evaluation of the dentin and marginal permeability after apicectomy as well as surface treatment are of great importance in successful apicectomy (3).

Removal of smear layer has been a controversial subject for years. Some authors reported that the presence of smear layer delays but does not eliminate disinfection (4), and acts as a beneficial barrier. This prevents microorganisms entering into the dentinal tubules, through inter-appointment contaminations. Other researchers suggested that the smear layer acts as a barrier which blocks the irrigation solution entrance into the dentinal tubules. It is suggested that removal of the smear layer helps the apical seal during retro-cavity preparation. Matsouka *et al.* observed a significant reduction of debris in the apical portion of the root canal using laser irradiation (5-6).

The appropriate interaction between retro-filling material and cavity walls depends not only on the characteristics of the material, but also on the superficial conditions of the cavity wall at the time of retrograde filling. Removal of debris and smear layer decreases the microleakage into the retro-cavity preparation (7); this provides a proper interface and better adaptation of the material to root end cavities (8).

Organic acids, ultrasonic instruments and lasers are being used to remove smear layer in endodontic treatments, but none are completely effective (9).

Application of materials including EDTA 17%, NaOCl 5.25% and Citric acid and MTAD were evaluated in different studies; but reported somewhat contradictory results (1,10). Takeda *et al.* showed that irrigation with EDTA 17%, phosphoric acid 6% and citric acid 6% did not remove all the smear layer from the root canal system, but CO_2_ laser was useful in removing and melting the smear layer on the instrumented root canal walls (11). In their other study Er:YAG laser was the most effective in removing the debris and smear layer from the root canal walls (12).

The preparation of root-end cavities by Er:YAG laser and ultrasonic showed lower values of microleakage in the lased group irrespective of the root-end filling material (13).

Some studies have shown that CO_2_ Nd:YAG, Argon, Er,Cr:YAG and Er:YAG laser irradiation can remove debris and smear layer from the instrumented root canal walls. Better removal of debris and smear layer has been reported by using an Er:YAG laser (12,14,15). Although residual debris could be found in the areas through which the laser light was unable to contact, it has been shown that in comparison with Nd:YAG, Er:YAG was more effective in removing debris and smear layer (16). Er,Cr:YSGG laser is very similar to the Er:YAG laser, they are called Erbium family (17).

A study on ultra morphological and chemical changes in Er,Cr:YSGG laser has shown that Er,Cr:YSGG laser irradiation resulted in partial or total removal of the smear associated with a few small regions of thermal injury, including carbonization and partial melting. Proper seal can be achieved by dentin irradiation using Erbium, chromium: yttrium-scandium-gallium-garnet (Er,Cr:YSGG) laser, which results in debris and smear layer removal as well (18). An SEM study revealed that the lased cavity surfaces which were prepared with Er,Cr:YSGG were irregular, and there was also an absence of smear layer while the dentinal tubules were exposed (19). Another study by Yamazaki *et al.*, indicated that Er,Cr:YSGG laser irradiation with cooling water spray is a useful method for removal of smear layer and debris (20). The present investigation was designed to evaluate dye penetration in the apical dentin after retrograde cavity preparation using Er,Cr:YSGG laser.

## MATERIALS AND METHODS

This study was conducted on the twenty-four mandibular permanent teeth with closed apices, collected and stored in normal saline prior to experiment. Only single-rooted teeth that were confirmed by radiographic views were included in this study. Root canals were debrided with Gates Glidden drills # 1, 2 and 3 (Dentsply/Maillefer, Ballaigues, Switzerland) in order to standardize canal access. The working length was established 0.5 mm shorter than the apical foramen. The root canals were instrumented up to HERO 642 rotary-file (Micro-Mega, Besançon, France) size #35 and irrigated with NaOCl 2.5% (Golrang, Tehran, Iran) at each file change. The canals received NaOCl 2.5% and normal saline as final irrigation. Canals were then dried with paper points followed by obturation with gutta-percha (Ariadent, Tehran, Iran) and AH26 canal sealer (Dentsply DeTrey, Konstanz, Germany). After 24 hours, all roots were resected from the 3 mm apical level using a fissure bur (Dentsply/Maillefer, Ballaigues, Switzerland) perpendicular to long axis of the tooth accompanied with a high-speed handpiece and water coolant.

Retrograde cavities were also shaped with a high-speed fissure bur (Dentsply/Maillefer, Ballaigues, Switzerland) to 3 mm depth and 1 mm in diameter. Twenty-four specimens were randomly assigned to 2 groups. In group I retrograde cavities were irradiated with Er,Cr:YSGG laser; while group II was not irradiated as the control group.

**Table 1:** Mean marginal infiltration in mm (±SD) of methylene blue dye. (n=24)

Group	n	Mean ( ± SD)	Median	Range of variables
**Er,Cr:YSGG laser** **(GI Test group)**	24	0.8 (±0.53)	0.65	.209-1.62
**Unlased (GII) **	24	0.97 (±0.54)	1.06	.47-1.14

The laser type used in this study was a Waterlase Millennium Er,Cr:YSGG laser system (Biolase Technology Inc., San Clemente, CA) with a wave length of 2.78 µm, pulsed with duration between 140 up to 200µsec, output power of 2 W, repetition rate of 20 Hz and air pressure 24% level and water level 34%. Laser energy was delivered using G4 for 15 seconds.

The retrograde cavities of both groups were filled with amalgam. All surfaces except for cut ones were covered with blue wax; all samples were then immersed in methylene blue 2% for 48 hours. All teeth were bisected longitudinally and dye penetration in two sections were measured using a SZX9 stereo microscope b 2000 (Olympus Optical Co., Ltd., Tokyo, Japan) at ×20 magnification by two blind observers.

Kolmogrove-Smirnov test was used to confirm normal distribution of data, students t-test was used for statistical analysis. The significance level was set at 0.05. Also, the Leven's test was used for evaluation of equal variance assumption in 2 groups.

## RESULTS

Dye penetration in two sides of retrocavities in each sample was observed by two blind observers and four values were recorded for each sample. Although the laser group showed less amount of dye penetration (Figure 1), the average of these values did not show any significant difference when using variance analysis for repeated values (P=0.39, F=0.054). Furthermore, the interclass coefficiency between these four values was 0.9337 (P<0.001). This study showed that, dye penetration in the lased group was (0.81 ± 0.53) and (0.97 ± 0.54) in the unlased group. No statistically significant difference was observed between the two groups in terms of apical leakage (Table 1).

## DISCUSSION

It has been shown that the dentin of apically resected root is more permeable to fluid than intact root; so reducing or eliminating the permeability of resected apical dentin would seem advantageous in root-end surgeries (21). Removal of debris and smear layer decreases the microleakage into the retro cavity preparation (7).

Better removal of debris and smear layer has been reported using an Er:YAG laser (12,14,15,19). To date, there has been different type of lasers including CO_2_, Nd:YAG, Er:YAG And Er,Cr:YSGG which have been used in endodontic treatments in order to facilitate and expedite the rate of disinfection and sterilization process, pain control following RCT, removal of debris and smear layer following root canal instrumentation (22,23).

It has been indicated that Er,Cr:YSGG laser irradiation is a useful method for removal of smear layer and debris from the root canal (20,24). The investigation by Altundasar *et al.* indicated that the Er,Cr:YSGG laser irradiation can remove smear layer partially or completely (18); comparing this study with our investigation implies other probable factors in smear layer removal.

However a study conducted by Kivanc *et al.* which shows that Er:YAG laser beam is not effective in removing debris and smear layer (25). According to this study, Er,Cr:YSGG laser can remove the debris and smear layer and consequently reduces the amount of dye penetration, though this was not statistically significant.

The results of the present study is close to Althundasar’s study which shows Er,Cr:YSGG laser can partially remove debris and smear layer. Such results may be due to the limitations of laser use in root canal system. Because laser is emitted in a straight direction, it is very difficult to irradiate the lateral canal walls (26). The size of the laser tip and duration of irradiation may be another influencing factor. It has been shown that by increasing irradiation time and using a wider tip size in order to scan the total surface areas, better results would be achieved (18). In this investigation, similar to a study conducted by Arisu *et al.*, the root ends were resected perpendicular to the long axis of the tooth in order to produce a reliable reference area for dye penetration measurement (27).

The standard deviation values of dentinal dye penetration in retrograde cavities were relatively high which indicates scattered distribution of data in both groups. This factor can explain why a significant difference was not observed between the two experimented groups.

Moreover, looking at such mixed results in apical leakage studies, it is necessary to pay special attention to methodological issues. Source of variation in the methods of study can be due to using different laser types, powers and modes, different settings (*in-vivo*, *in-vitro* conditions), target tissues, irrigation solution as well as different dye and obturation materials.

## CONCLUSION

In the condition of this *ex vivo* study, it was shown that dye penetration was less in lased group. This is due to better seal of the dissected surfaces; however, the difference between the two experimented groups was not statistically significant; further investigations are recommended.
